# Corporate social responsibility, policy framing and strategic marketing: understanding the alcohol industry’s use of social media in Uganda

**DOI:** 10.1186/s13011-024-00611-z

**Published:** 2024-06-20

**Authors:** Matthew Lesch, Su Golder, Jim McCambridge

**Affiliations:** 1 Department of Politics and International Relations, Derwent College, University of York, York, YO10 5DD UK; 2 Department of Health Sciences, Seebohm Rowntree Building, University of York, York, YO10 5DD UK

**Keywords:** Alcohol policy, Alcohol industry, Corporate social responsibility, Uganda, Social media

## Abstract

**Background:**

Sub-Saharan Africa is important to the future of alcohol and global health because the alcohol market there is expanding rapidly in a relatively young population. This entails a corresponding contest about whether the policy measures adopted will be shaped by scientific evidence or by industry interference in alcohol policy. This study examines how alcohol industry actors use social media.

**Methods:**

Uganda was selected for study because of high levels of alcohol harm and recent alcohol policy debates. Data on the X (formerly Twitter) activity of the Ugandan companies of AB InBev and Diageo, who are the two main brewers, and the trade association including both, were collected, coded and thematically analysed.

**Results:**

X is used overwhelmingly by alcohol industry actors in Uganda to promote corporate social responsibility (CSR) and alcohol policy framing content. There is little direct product marketing. The framing of policy problems and solutions, and of the actors involved in policymaking and CSR resembles that used elsewhere in the political strategies of the transnational alcohol corporations. Content which appears more emphasised in Uganda includes material on farmers, illicit trade and contribution to the economy. As elsewhere, it avoids giving attention to the policy measures which would make a difference to the levels of alcohol harms endured by Uganda. Rhetorically, X is thus used to create a parallel universe, in which the actual harms and what is known about how to reduce them are conspicuous by their absence.

**Conclusions:**

The alcohol industry presents itself as indispensable to Uganda’s future and appears to have developed relationships with politicians, partnerships with government, and built a coalition with farmers. This means the alcohol industry may be well positioned to oppose public health policy measures, even though their arguments lack substance and are at odds with the evidence.

## Background

Alcohol consumption presents a serious challenge to global health, increasingly so in the developing world. Studies have demonstrated the role of the alcohol industry in resisting public health policies [1, 2]. Much of this work has tended to focus on industry activities within developed countries [3, 4], reflecting the burden [5, 6], and studies of alcohol-related harm and the political activities of the alcohol industry across a wider range of contexts are necessary [7–9].

Sub-Saharan Africa is recognised for having weak alcohol policies and as a result, there have been sharp increases in the accessibility and affordability of alcohol [10]. Low-income countries and emerging markets are a key strategic focus for transnational alcohol producers [7, 11]. A small number of studies have already documented the activities of global alcohol producers in the African region where the population is young compared to other regions [12, 13]. The alcohol industry successfully shaped alcohol policies in four sub-Saharan African countries: Lesotho, Malawi, Uganda, and Botswana to avoid the measures that evidence shows can be expected to be effective [3]. Transnational alcohol corporations, such as Diageo, AB InBev, and Heineken, have pushed for open markets and minimal government intervention. On the other hand, the World Health Organization (WHO), aided by civil society organisations, has worked to guide governments in evidence-based approaches to alcohol harms [11]. Post-colonial governments have often sought to promote economic growth through industrial expansion, with limited appetite and capacity for regulations [10].

Uganda stands out as one of the African countries with the highest per capita alcohol consumption rates, leading to a range of adverse health and social outcomes. In 2004, Uganda had the highest annual alcohol consumption globally. Even though the WHO has reported a decline in Uganda’s per capita alcohol consumption, it remains one of Africa’s top alcohol-consuming countries [14]. Population –level data on the distribution of alcohol consumption in Uganda is very limited. The data on alcohol consumption patterns show that heavy episodic drinking is more common among males (68.8%) than females (32.6%) [14]. Additionally, it has been estimated that 11% of the total alcohol consumed is beer, 3% spirits, less than 1% is wine, with the remainder likely to consist of other beverages such as homemade fermented drinks [14]. Studies have found that Uganda has one of the highest prevalence of negative consequences from alcohol [15]. More recent studies also show high rates of hazardous and harmful consumption among those who drink alcohol, and as elsewhere, without clear gender differences among young people [16]. Public health actors in Uganda have drawn attention to high levels of alcohol harm. The Uganda Alcohol Policy Alliance (UAPA), for example, brings together different civil society actors committed to reducing alcohol related harm in Uganda through advocacy for effective, evidence-based policies [17]. UAPA has also drawn particular attention to the influence of the alcohol industry [12, 18]. The alcohol industry’s role in policymaking in Uganda is poorly understood.

Analysis of data that exists in the public domain, including social media data, has the potential to enrich understanding of industry actors and their activities. Transnational companies use several different platforms to market their products. In recent years, this has involved using X and other forms of social media [19]. In addition to marketing, however, social media can have a major influence on policy debates, including public health issues [20, 21]. Existing studies indicate that industry groups use X to re-frame policy issues to advance their goals [22, 23]. This involves portraying themselves as authoritative and credible figures in public health policy and/or deflecting potential concerns about their product and impacts through re-framing [24]. For example, alcohol industry actors often tout their corporate social responsibility (CSR) credentials [25]. CSR activities refer to an organisation’s claimed efforts to contribute towards the resolution of major societal problems such as poverty and under-development, public health and/or environmental issues. Such claims do not preclude that activities may also be influenced by other motivations (e.g., reputation management), which thus may also serve public relations goals (e.g., greenwashing) [26, 27]. This means that convergence and synergies can occur between CSR, marketing, and political strategies, which may be co-ordinated [2].

The present study addresses two key questions. First, what are the main issues that the key alcohol industry actors in Uganda concentrate on in social media communications? Second, based on these data, what insights are possible into the goals and strategies pursued by the principal alcohol industry policy actors in Uganda?

## Methods

We first oriented ourselves to the political context [28–31] and alcohol policy context in Uganda [32–34]. Second, using Google searches, we identified several prominent alcohol industry policy actors. We then used X to identify accounts for Uganda Breweries Ltd, Nile Breweries Ltd, and the Uganda Alcohol Industry Association (UAIA).

Uganda Breweries is one of the largest and oldest beer producers in Uganda. The company was started in 1946, as part of East African Breweries Limited (EABL), a Kenya-based company. Diageo, one of the world’s largest alcohol producers, acquired majority control of EABL in 2000 [35], and as well as beer, the company produces spirits, including vodka and whisky, with both Ugandan and international brands. Nile Breweries is the other major beer producer in Uganda. The brewery was started in 1951 and in July 2001, SABMiller (now AB InBev) acquired full ownership of the company. Niles Breweries controls 59% of the beer market in Uganda [36].

The UAIA is a trade association representing the interests of alcohol producers in Uganda. It was established in 2006 to “ease engagement, collective lobbying with government agencies on issues of mutual interest” as well as to promote “responsible use and marketing of alcoholic beverages” [37]. The leadership team comprises senior executives from Nile Breweries and Uganda Breweries.

Using Mozdeh software, we downloaded all publicly available posts from these actors’ accounts. Restrictions governing X data use limited the number of posts we could collect. As such, we collected posts from a specified time range and excluded reposts and non-English language posts (see Table 1). In total, we collected 2504 posts.

**Fig. 1:**
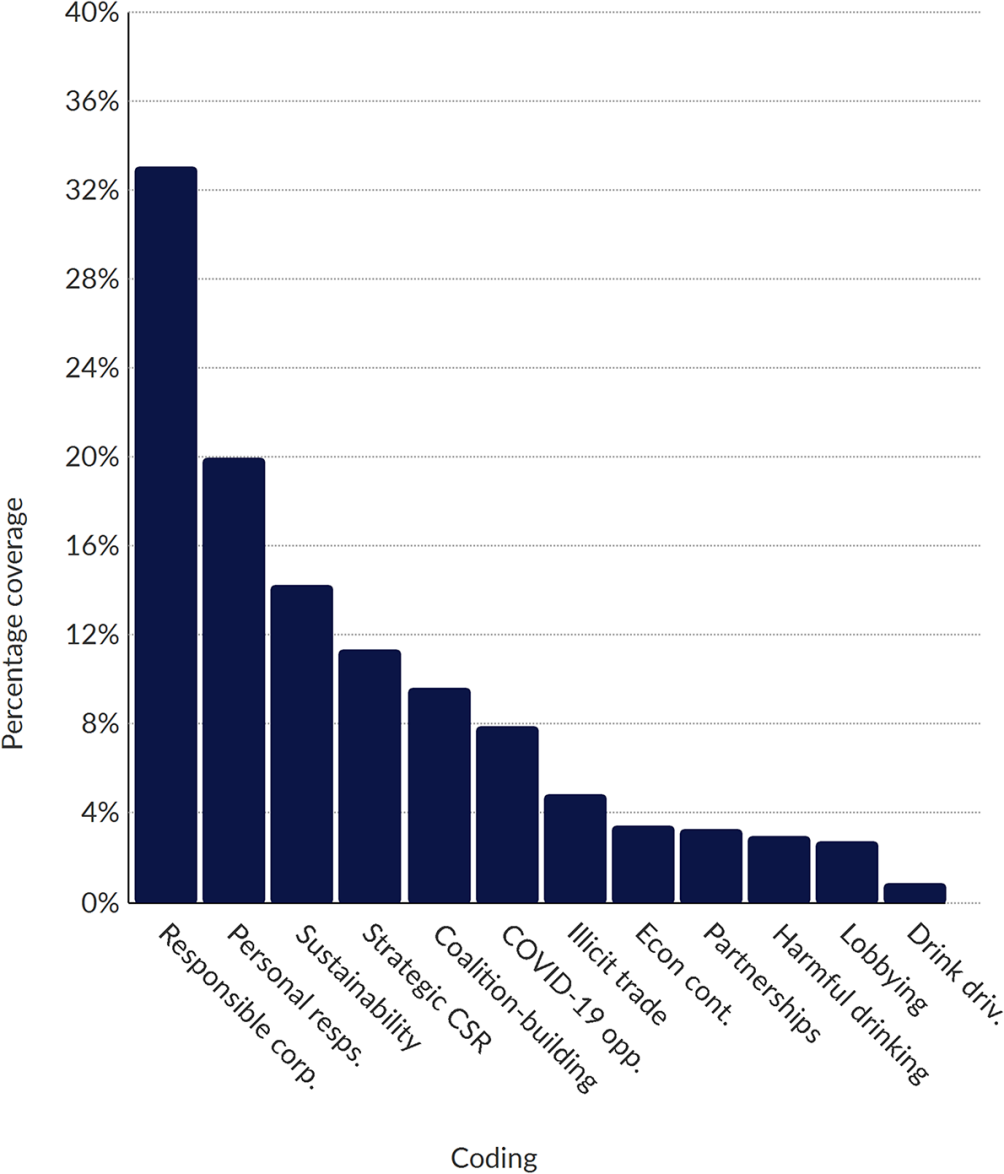
Proportion of sub-theme Coding for Niles Breweries (AB InBev). *Note* The bars in the chart illustrate the distribution of sub-theme coding for Niles Breweries. Full names for each sub-theme, including descriptions, are provided in Table 3

**Fig. 2:**
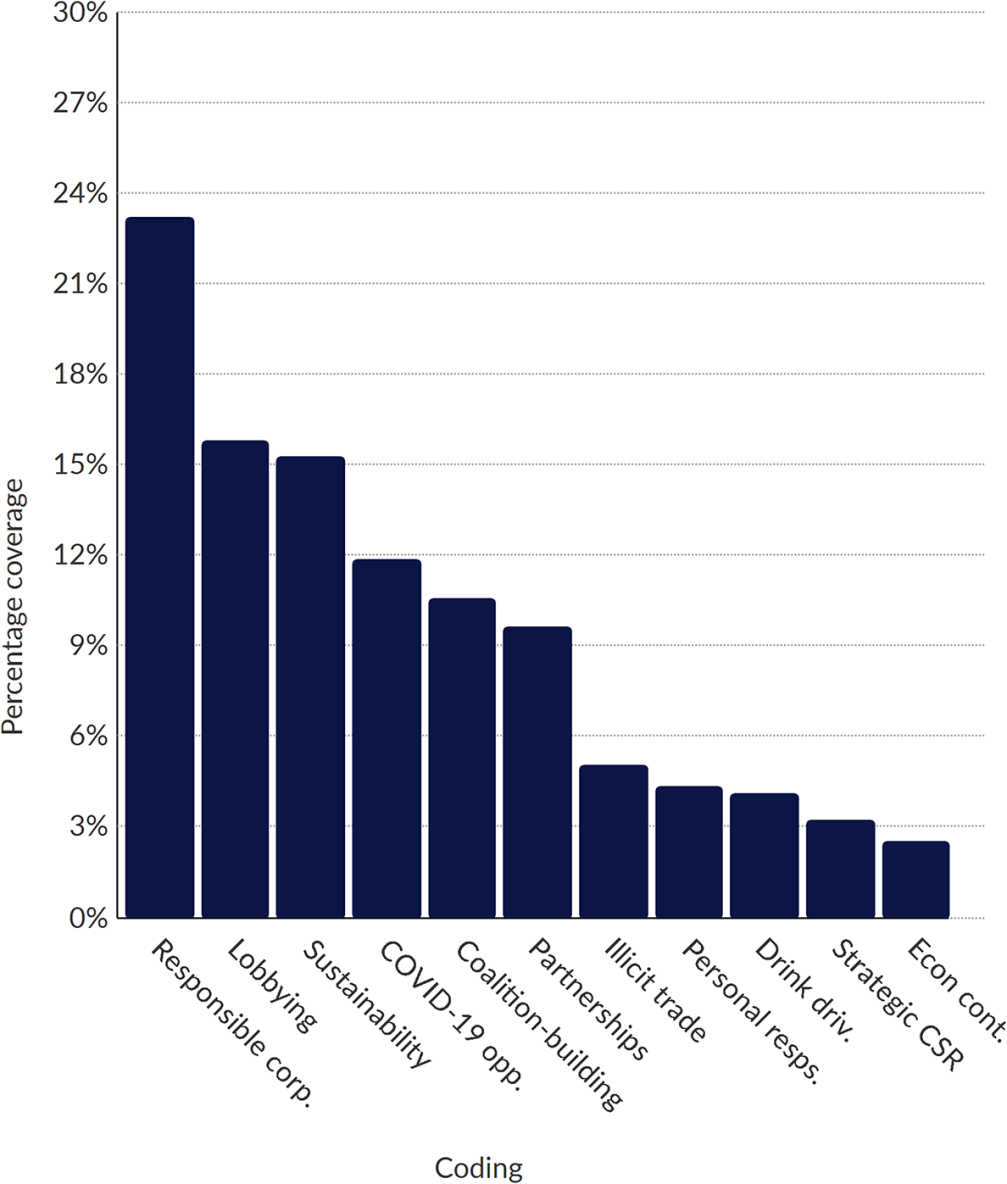
Proportion of sub-theme coding for Uganda Breweries (Diageo). *Note* The bars in the chart illustrate the distribution of sub-theme coding for Diageo. Full names for each sub-theme, including descriptions, are provided in Table 3

**Fig. 3:**
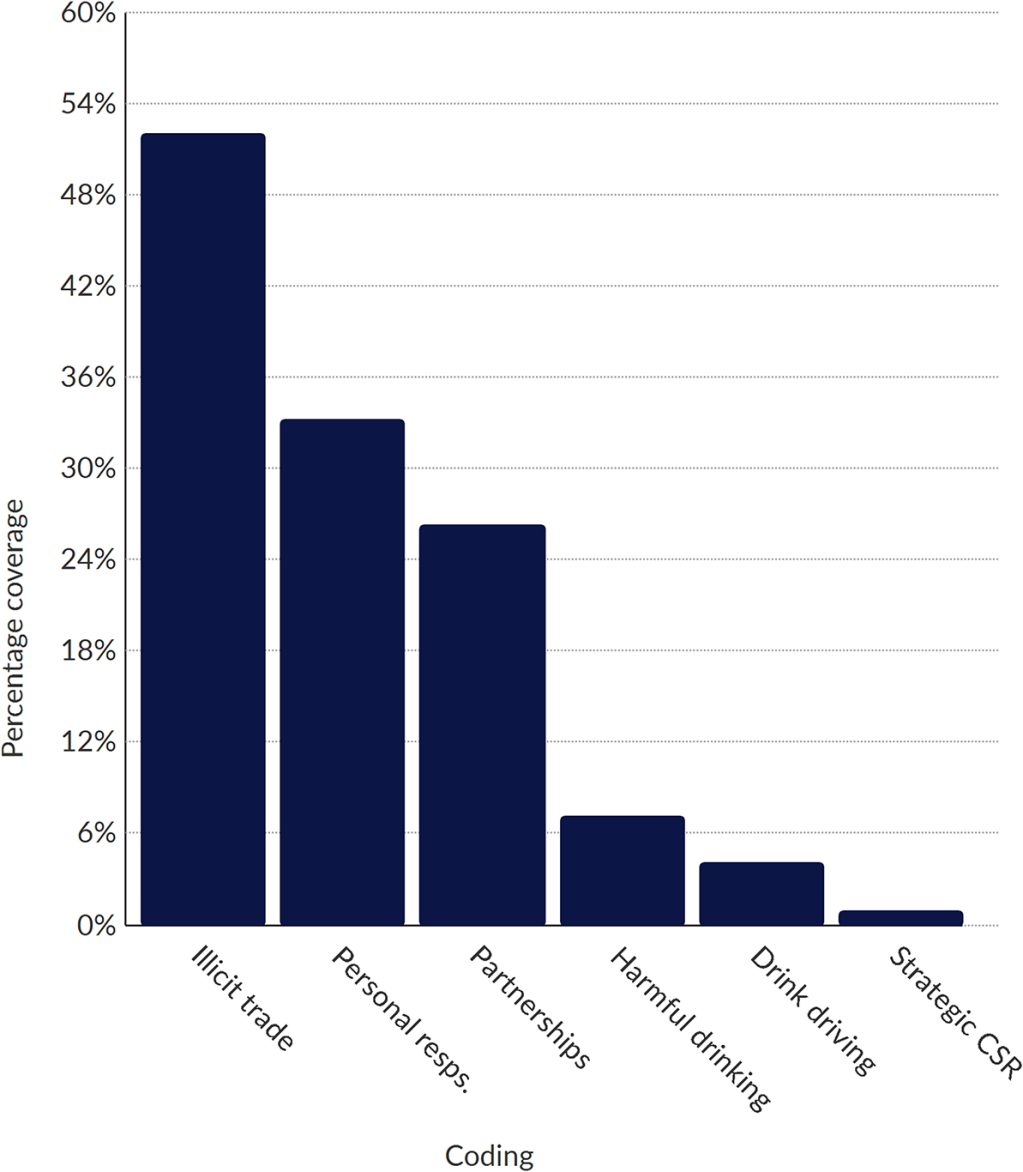
Proportion of sub-theme coding for Uganda Alcohol Industry Association (UAIA). *Note* The bars in the chart illustrate the distribution of sub-theme coding for UAIA. Full names for each sub-theme, including descriptions, are provided in Table 3

Posts were then placed into NVivo software, in which we performed a thematic analysis of the data [38]. Using a combination of deductive and inductive techniques, we coded and analysed the entire dataset. Deductively derived codes (see Table 2) were informed by previous research on alcohol industry activities, including literature on policy framing and corporate social responsibility activities [1, 25, 39]. Inductively generated codes were developed as sub-themes; our sub-themes were refined and emerged iteratively throughout the coding and analytic process (e.g., sustainability-related CSR), and could contribute to more than one main theme. Figures 1, 2 and 3 (see below) summarise coding results by industry actor. The results of our thematic analysis are reported below.

**Table 1:** Data source summary

Actor	X Handle	Date Joined X	Total number of posts	Posts down-loaded	Date range
Uganda Breweries Limited	@UgandaBreweries	March 2015	8,475	902	4 Nov 2021 – 3 April 2023
Nile Breweries Limited	@NBLUganda	August 2016	2,559	1532	28 Aug 2016 – 3 Apr 2023
Uganda Alcohol Industry Association (UAIA)	@uaia_ug	April 2022	129	70	4 Apr 2022 – 3 April 2023

## Results

Across the dataset, our analysis supported several identified core themes (see Table 2) and numerous sub-themes (see Table 3). Overall, industry actors’ posts fell into three broad and interlinking categories. First, industry actors used X to promote their products to consumers (i.e., marketing), though much less than other activities (see Table 2). Second, X was used to portray the alcohol industry as a vital and socially responsible part of the Ugandan economy and society (i.e. CSR). Third, industry actors used social media to advance their policy-relevant interests by framing alcohol policy problems in particular ways, as well as promoting specific policy solutions (e.g., self-regulation). Finally, industry actors identified specific stakeholders and policy actors (see Table 2), most prominently Ugandan farmers (see Table 3).

**Table 2:** Core themes

Code	Description	Coding frequency	Percentage of dataset
Corporate social responsibility (CSR) [25]	Posts focusing on alcohol industry actors’ CSR activities.	883	46.16%
Alcohol as a policy problem [1]	Posts focusing on efforts to address the negative health, economic, and social impacts of alcohol consumption.	367	19.18%
Alcohol policy actors [1]	Posts identifying the industry’s interactions and/or relationships with other key alcohol policy stakeholders.	337	17.62%
Alcohol policy solutions [1]	Posts specifying policy solutions designed to address alcohol-related problems.	243	12.7%
Product marketing [40]	Posts marketing or advertising alcohol products to consumers and/or retailers.	83	4.34%
**Total**		**1913**	**100%**

### CSR

Corporate Social Responsibility (CSR) constituted a clear priority for alcohol industry actors in Uganda. These actors extensively employed X to spotlight their CSR initiatives. The CSR activities encompassed various topics and implied diverse strategies. The overarching strategy central to CSR posts, however, seemed to be positioning the alcohol industry as a responsible corporate citizen (*n* = 333).

The content and context of CSR posts varied considerably. Many of these posts specifically addressed the topic of alcohol consumption, promoting responsible drinking, for example. Other efforts, however, seemed designed to portray the alcohol industry as contributing members to the Ugandan and global community.

#### CSR and responsible drinking campaigns

A significant portion of the CSR posts described specific campaigns launched by the alcohol industry to reduce “harmful drinking” or promote “responsible drinking.” These posts often included hashtags such as #Responsibledrinking and #BeSmartDrinkSmartUg. For instance:

*@NBLUganda: We are launching our Be Smart, Drink Smart campaign today today … It is aimed at promoting a culture of responsible drinking and reduce the harmful use of alcohol. #BeSmartDrinkSmart*.

Industry actors did not offer clear definitions of these concepts. For example, it claimed that “drinking responsibly… is largely a subjective matter.”

Alcohol industry actors highlighted CSR campaigns targeting specific populations, such as students:

*@NBLUganda: Our Country Director, David Valencia is a big advocate for smart drinking. Join him in making a better world by participating in the Inter-University Smart Drinking Challenge… #BeSmartDrinkSmartUg*.

Such campaigns imply that “harmful drinking” is predominantly an issue among young people or specific minority groups. This framing suggests that population-level measures may not be necessary, given that problematic drinking patterns may be primarily confined to certain groups.

Responsible drinking CSR posts did not always refer to specific campaigns. In other instances, industry actors used X to encourage consumers to adopt particular behavioural strategies regarding drinking, so that X itself was the CSR vehicle. For example:

*@UgandaBreweries: Eat something before you start drinking. Space your alcohol with water or soft drinks. Don’t drink and drive. Take a cab back home or get a designated driver to take you home. @RedCardUganda*.

#### Sustainability-related CSR

CSR activities were not limited to alcohol consumption. CSR serves as a crucial vehicle for demonstrating an organisation’s alignment with the values of the community it operates within. As such, sustainability-related issues constituted a common CSR topic for the industry (*n* = 169). Industry actors promoted specific environmental initiatives, policies, and partnerships. These range from planting 40 million trees (Uganda Breweries) to constructing boreholes in rural areas (Niles Breweries). Sustainability-related CSR posts often highlighted partnerships with government departments (e.g., Ministry of Water and Environment and local municipalities) and civil society groups (e.g., World Wide Fund for Nature).

Industry actors also employed social media to underscore the alignment of their values (e.g., sustainability) with their business practices:

*@NBLUganda: Without water, our business cannot operate effectively and the livelihood of 1000s would be heavily affected. Water contributes to a tune of 90% of every hectoliter of beer we produce. We consider water as one of the major driving force of sustainable development. #WorldWaterDay*.

#### COVID-19 as an opportunity for CSR

A related strategy was to identify strategic opportunities to emphasise its commitment to society. During the onset of COVID-19, for example, alcohol producers leveraged opportunities to enhance their CSR credentials. Alcohol producers funded COVID-19 vaccination sites and frequently highlighted their contributions to containing the virus on social media.

*@NBLUganda: Nile Breweries Limited, alongside the @MinofHealthUG, have set up 20 #Covid19 vaccination centres at NBL distribution sites across the country. We’re proud to have been able to support Uganda’s healthcare system and its brave health workers in these difficult times. #PlayYourPart*.

#### Other CSR-related issues

Financial and/or organisational support for various health initiatives, including blood donation drives and HIV/AIDS fundraisers, were also consistently highlighted. Finally, the studied actors were highly strategic in their use of social media (*n* = 89), linking their organisations to specific developments within society. For example, alcohol producers frequently highlighted CSR activities in relation to different national and global celebrations. These included International Women’s Day, World Water Day, World Disability Day, International Day for Tolerance, Uganda Water and Environment Week, Labour Day, World AIDS Day, World Safety Day, World Food Safety Day, and World Blood Donor Day.

**Table 3:** Description and frequency of sub-themes

Sub-theme	Core theme	Description	Coding frequency	Percentage across entire dataset
Responsible corporate citizens	CSR	Presenting the alcohol industry as contributing to the betterment of society, beyond producing alcohol.	333	23.04%
Personal responsibility	Alcohol as a policy problem CSR Policy solutions	Stressing the importance of individuals’ drinking habits and behaviours in understanding alcohol-related harms.	201	13.91%
Sustainability-related CSR	CSR	Highlighting specific CSR initiatives that focus on sustainability and/or environmentalism.	169	11.7%
COVID-19 as an opportunity for CSR	CSR	Highlighting the alcohol industry’s financial support for responding to COVID-19.	151	10.45%
Coalition-building with farmers	Alcohol policy actors	Describing efforts to support and promote the economic interests of farmers in Uganda.	115	7.96%
Strategic CSR	CSR	Seizing domestic and global developments as opportunities to highlight CSR credentials.	89	6.16%
Illicit trade	Alcohol as a policy problem	Focusing on illicit trade in Uganda as a priority for reducing alcohol-related harm.	88	6.09%
Contribution of the industry to the economy	Alcohol policy actors CSR	Focusing on the importance of the alcohol industry to the economic health of Uganda.	88	6.09%
Partnerships with the Uganda government	Alcohol policy actors	Stressing the close working relationship between the alcohol industry and different parts of the Ugandan government.	82	5.67%
Lobbying government officials	Alcohol policy actors	Identifying key interactions between alcohol industry actors and government officials in the context of Ugandan alcohol policy.	57	3.94%
Harmful drinking	Policy problem	Describing a specific pattern of alcohol consumption in which individuals are more likely to experience harm.	37	2.56%
Drink driving	Policy problem CSR	Identifying drink driving as a pressing alcohol-related issue to address.	35	2.42%
**Total**			**1445**	**100%**

Proportion of Sub-theme Coding for Niles Breweries (AB InBev) – the bars in the chart illustrate the distribution of sub-theme coding for Niles Breweries. Full names for each sub-theme, including descriptions, are provided in Table 3.

Proportion of Sub-theme Coding for Uganda Breweries (Diageo) – the bars in the chart illustrate the distribution of sub-theme coding for Uganda Breweries. Full names for each sub-theme, including descriptions, are provided in Table 3.

Proportion of Sub-theme Coding for Uganda Alcohol Industry Association (UAIA) – the bars in the chart illustrate the distribution of sub-theme coding for UAIA. Full names for each sub-theme, including descriptions, are provided in Table 3.

### Alcohol as a policy problem

In addition to CSR, alcohol industry actors used social media to frame alcohol harms. The issues stemming from alcohol-related matters, however, were frequently presented in ways that aimed to safeguard the economic interests of producers.

#### Responsible drinking

A personal responsibility narrative frames the problem as the individual drinker, rather than the product or the producer. Alcohol harms were consistently depicted as a result of alcohol misuse by irresponsible individuals (*n* = 201):

*@uaia_ug: …alcohol is not bad. The bad thing, however, is the abuse of alcohol under which underage drinking falls. Parents need to be sensitised about how their own drinking influences their children. #ResponsibleDrinking*.

#### Harmful drinking

Linked to responsible drinking, alcohol industry actors acknowledged some of the problems arising from alcohol consumption mainly through the lens of harmful drinking (*n* = 37). Much of the messaging around responsible drinking also references harmful drinking as the converse:

*@NBLUganda: We want to ensure a long-term and sustainable reduction in harmful drinking by empowering consumer behaviors and the need to shift- consumers decisions to make better smart drinking choices. #BeSmartDrinkSmart @f_aduk*.

In another tweet, Niles Breweries identified the need to cultivate a better drinking culture:

*@NBLUganda: We have a unique role to play in fostering a smart drinking culture – not only today, but every day. Proud to be a #SmartDrinkingChampion. #DrinkSmartToday…*.

UAIA often tries to distinguish those who drink alcohol from those who have problems with alcohol:

*@uaia_ug: “… let us separate Alcohol consumption & Alcohol Abuse because as a business we are never proud when alcohol is abused and the Responsible code is aimed at ensuring that the products are not abused by consumers and manufacturers.” - @onapitoEkomolo2*.

#### Illicit trade

Illicit trade was regularly tweeted about as one of the main contributors to alcohol-related harm (*n* = 88), alcohol-related health risks identified:

*@UgandaBreweries: Every Ugandan should be at the forefront of fighting illicit trade because not only does it affect our revenues, it also endangers health. Illicit alcohol, for instance, kills almost instantly…*.

Some posts focused on the economic ramifications of the illicit market, highlighting that the government loses substantial revenue due to this market:

*@UgandaBreweries: “We have done research on illicit alcohol and realized that @URAUganda is only getting 35% of what they should be getting from the alcohol sector. The gov’t is missing over UGX 4 trillion in tax in our industry alone”…*.

#### Drink driving

Industry actors rarely acknowledged the most recognised harms associated with alcohol consumption, with the noteworthy exception of drink driving (*n* = 35). Some of these efforts took the form of specific CSR campaigns. For example, during the holiday season, Niles Breweries encouraged consumers to pledge that they would not engage in drink driving:

*@NBLUganda: Enjoy responsibly. Do not drink and drive. Show your commitment by signing the pledge*: http://togetherforsaferroads.org/tsr-pledge/*#SaferRoads*.

Drink driving was also a common policy concern for UAIA. In introducing the association’s self-regulatory code of conduct, for instance, UAIA drew attention to what it perceived to be key alcohol policy issues:

*@uaia_ug: “This code does not serve to summarize or substitute national laws, and policies, which must always be upheld but rather explain UAIA’s approach to self-regulation & address social issues such as drinking & driving, underage drinking, & heavy episodic drinking”- @onapitoEkomolo2*.

The focus on these specific alcohol policy issues was prevalent in UAIA posts, suggesting that this is what alcohol industry actors wish policymakers and the public to concentrate on. For example:

Efforts to curb drink driving sometimes formed the basis of CSR campaigns initiated by alcohol producers.

*@UgandaBreweries: The #WrongSideOfTheRoadUG campaign is part of our Society 2030: Spirit of Progress agenda. It is part of our efforts to Promote Positive Drinking by helping people understand effects of drink driving from real people & real stories. Be part of it!*

#### Alcohol and health impacts

In the limited number of posts where health consequences were acknowledged, these often focused on alcohol’s short-term rather than long-term health effects. For example:

*@NBLUganda: Beer tends to have a dehydrating effect on your body. Drinking water between your drinks can counter this effect, preventing hangovers, and making it a great experience. #SmartDrinkingWeek #ArtOfDrinking #GBRD22 #BeSmartDrinkSmart*.

#### Alcohol policy actors

A significant proportion of the social media activity is devoted to framing the function, roles, and responsibilities of various alcohol policy actors, including the industry itself (*n* = 337). In many instances, social media was used to identify groups with which alcohol industry actors were collaborating on issues to which they wished to draw attention.

#### Coalition-building with farmers

One clear priority was to showcase the strength of the alcohol industry’s relationships with Ugandan farmers (*n* = 115). Farmers and the broader agricultural community in Uganda are frequently portrayed as indispensable and key strategic partners. Farmers were often used as political symbols, designed to indicate the alcohol industry’s support for a crucial constituency in Uganda. For example:

*@NBLUganda: Farmers are our true heroes. We intention to support local farmers in Uganda by creating a ready market for their produce. Your favourite beers are made from homegrown materials Barley, Sorghum, Cassava & Maize. #FarmerLiveihoods #QualityBrew #BetterWorld*.

Farmers’ wellbeing was regularly identified as a key concern. For example,

*@NBLUganda: The partnership with @narouganda is a continuation of our commitment local sourcing and improving livelihoods of Ugandan Farmers. With the current local sourcing at 98%, annually, NBL purchases around US$23 M worth of produce from local farmers.#SmartAgriculture #BetterWorld*.

*@UgandaBreweries UBL works with over 35,000 farmers who directly impact close to 70,000 households. Our partnership is boosting farmers’ productivity, improving their household income and promoting the Buy Uganda Build Uganda (BUBU) initiative at the agricultural sourcing level. #UBLGrowingUganda*.

Associations of this nature reveal the interconnections between the industry’s efforts to engage in coalition-building, CSR, and informal lobbying (see below).

#### Contribution to the economy

As noted above, efforts aimed to underscore the sector’s contribution to the Ugandan economy (*n* = 88), including as taxpayer and employer. For example:

*@UgandaBreweries: “Last year, UBL was the third top tax payer in the country. Thank you for being good partners. As @URAuganda, we are committed to getting closer to you, to listening to you and walking together for the development of our country.” - @URA_CG #UBLGrowingUganda*.

*@UgandaBreweries: “…we have structured our business in a way that we truly share prosperity & value with Ugandans. This includes supporting @GovUganda aspirations such as BUBU, tax contribution, employment and other environmental, social and governance impacts. - @Kilonzo_Andrew #UBLGrowingUganda*.

The major brewers used X to highlight societal recognition of their economic and social impacts:

*@NBLUganda: “Secondly, I want to thank NBL for being one of the top tax payers. I’m being told that last year they contributed over Ush400 billion to the tax effort. Without taxes running the economy is almost impossible.” ~ Hon. @MatiaK5 #NBLEconomicForum*.

#### Partnerships with the Ugandan government

Alcohol industry actors also used X to emphasise significant partnerships with the Ugandan government (*n* = 82). These partnerships often took the form of joint activities or funding:

*@NBLUganda: We joined trade partners and the Ministry of Water &. Environment –South Western Region, to mark Uganda Water and Environment week 2021. The team cleaned the banks of R. Rwizi at Rwebikoona village, Mbarara which is choking with plastic waste. #WaterStewardship #WWD21*.

*@UgandaBreweries: “We celebrate corporates like Uganda Breweries for being businesses with a purpose. They find needs in the communities and utilize their resources to address them,” - Dr. @DianaAtwine. Permanent Secretary @MinofHealthUG #UBLGrowingUganda*.

Alcohol companies were also portrayed by government officials as pivotal partners in delivering infrastructure projects to local communities:

*@UgandaBreweries: “I worked with UBL to improve sanitation in Nakawa during my time as Mayor & I am still working with them. I thank the brewery for making the people of Nakawa a priority in their operations & ask them to continue.” - @RonaldBalimwezo, MP Nakawa East. #UBLGrowingUganda…”*.

The willingness of the Ugandan government to partner with major alcohol companies on various health, social, and environmental initiatives likely reflects the government’s limited fiscal resources and policy capacity.

#### Lobbying government officials

The alcohol companies also highlighted the depth of their relationships with public officials (*n* = 57), including the Office of the President, the Ministry of Health, and the Ministry of Finance. The nature of these interactions was not always explicit, yet the frequency of contacts reported on X suggests that alcohol producers have well-established connections within the Ugandan government. Some of these activities mirror alcohol industry-government interactions in other countries. For example:

*@UgandaBreweries: A team from Uganda Breweries and @uaia_ug were at @Parliament_Ug as part of stakeholder consultation engagements to generate views for the drafting of the proposed Alcoholic Drinks Control Bill. They met with among others, the bill drafter Hon. Sarah Opendi.*

Industry actors, however, also gained access to government officials through one-off meetings:

*@UgandaBreweries: Earlier today, our [Managing Director] @Kilonzo_Andrew and a team from the brewery were at @mofpedU for a courtesy meeting with the Permanent Secretary/Secretary to the Treasury @rggoobi at the Ministry offices. [1/4]… The team met with Moses Kagwa, the Director Economic Affairs as well. [4/4]*

Government officials were often willing to express support for the alcohol industry. This sentiment was frequently highlighted by alcohol producers on social media. For instance, during an economic forum, Niles Breweries shared the following quote from the Minister of Finance, Matia Kasaija:

*@NBLUganda: “I encourage my (fellow) Ugandans to support, by buying NBL products, so that they can continue expanding.” ~ Hon. @MatiaK5. #NBLEconomicForum*.

Interactions with public officials were often used to reinforce specific problem-definitions and preferred policy solution frames. During consultations over changes to alcohol legislation, for example, Niles Breweries tweeted the following:

*@NBLUganda: This bill should focus on illicit alcohol, which is 65% of all the alcohol consumed in Uganda. Separate the illicit from the regulated alcohol…” [1/3]… We noticed in the proposed bill there’s a lot of restrictions, like the one on trading hours. We think this is anti-freedom. You’re limiting people’s ability to socialize, and yet Uganda is a land of freedom[3/3]*.

Interactions with government officials were not limited to Ugandan officials. Alcohol industry actors also met with foreign diplomats. For example:

*@UgandaBreweries: Eunice Waweru, the Ag. [Managing Director] of Uganda Breweries was today accompanied by our Corporate Relations Manager @tahakanizibwa to meet with Lord Popat, the British Prime Minister’s Trade Envoy to Uganda and Rwanda*.

Alcohol companies also had opportunities to strengthen relationships with government officials and other key policy actors in different informal settings. Uganda Breweries’ management, for example, was invited to the British High Commissioner’s residence in October 2022 to celebrate British-Ugandan relations. High-profile guests at this event included the Prime Minister of Uganda and several other key officials.

### Alcohol policy solutions

Across the four policy-related core themes, discussions of alcohol policy solutions were the least prominent, but still received substantial attention on social media. Two key developments are important for contextualising this thematic content. First, the Ugandan government approved a National Alcohol Control Policy in June 2019. The policy document identifies the scope of alcohol harms in Uganda, calls for strong regulation on production, availability, marketing and pricing, and the need for better research and monitoring of alcohol consumption patterns in Uganda [41]. Despite this, government leadership has been largely absent. Second, a private member’s bill, the Alcoholic Control Bill, was introduced in the Ugandan parliament by the Honourable Sarah Opendi in November 2022 (subsequently becoming the Alcoholic Drinks Control Bill, 2023 in November 2023). The legislation proposes several key public health provisions, such as advertising restrictions, limiting the hours for alcohol sales in bars to 10:00 pm on weekdays and midnight on weekends, prohibiting sales to those under 18 years of age, and key licensing restrictions on alcohol sales [42]. The legislation is currently undergoing scrutiny in various committees but has faced major opposition from alcohol industry groups [43–45].

Alcohol industry actors responded to these policy developments in a few different ways. Initially, the UAIA unveiled a “Mind Your Drink” public awareness campaign and associated political activity designed to curb the consumption of illicit alcohol. X was used to launch this campaign:

*@uaia_ug: we are dedicated to promote responsible drinking, self regulation of our members in advertising & to work with [different government departments] to eradicate illicit Alcohol on the market…*.

A common industry criticism is that weak enforcement is to blame for the illicit trade in Uganda and elsewhere. Alcohol industry actors used X to justify why addressing the illicit market, rather than introducing new population-level measures, should be the focus of policymakers. For example, UAIA tweeted:

*@uaia_ug: Illicit Alcohol Trade grows by 5% every year & this goes unchecked. There is a levy in place but enforcement is still lacking. We need to tackle the 70% of illicit trade before we think of new regulations because we have an issue before us that Is going unchecked.*

The alcohol industry also took other steps to dissuade policymakers from broadening the scope of alcohol control efforts. In February 2023, the UAIA proposed a new regulatory code of conduct for producers (i.e., self-regulation), claimed not to substitute for regulations:

*@uaia_ug_: …The UAIA Code goes to the root of our commitment in self-regulation. The code does not serve to summarize or substitute national laws, policies, which must always be upheld but rather explain UAIA’s approach to self-regulation*.

Posts from the UAIA also help illuminate the political context in which the code was developed. For example, as one industry insider explained in a speech to industry:

*@uaia_ug_: … “the Responsible Code is timely since we have to change the perspective of consumption because what has been portrayed is hurting the industry and the workers. because [the alcohol industry] is a big source of employment”*.

Finally, the brewers used social media to express their support for the code,

*@UgandaBreweries:“The #UAIAResponsibleCode will create safeguards for consumers by regulating distributorship, marketing consumption of alcoholic beverages to protect the alcohol industry’s reputation & freedom of expression in brand activities across all our alcohol brands.” - @JulzKagwa*.

### Product marketing

Although marketing to consumers was not as prominent as the CSR and other policy-related themes, it still constituted one component of social media strategy. References to specific products and/or ways to acquire these products were the most common use of alcohol marketing (*n* = 83). Alcohol companies promoted their products, while the trade association never engaged in any such explicit product marketing.

For example,

*@UgandaBreweries: The Baileys Delight is a light and lush cream liquor which blends the luscious taste of African honey with real dairy cream. For your taste buds and drinking pleasure, get yourself a 200 ml or 750 ml bottle from your nearest outlet.*

Alcohol marketing was somewhat intertwined with problem-definition and CSR thematic content, as product advertisements were commonly accompanied by messages of “drink responsibly” (Diageo/Uganda Breweries) or “smart drinking” (AB InBev/Nile Breweries):

*@UgandaBreweries: Midweek enjoyments with friends? Get the Johnnie Walker Black Label 1 L from Party Central by UBL at just Shs 131,600. Enjoy our unmistakably smooth, impressive whisky from Scotland. Drink Responsibly.*

There were also clear connections between direct product marketing and other kinds of CSR content. While advertising its cider, for instance, Uganda Breweries highlighted its commitment to Ugandan farmers:

*@UgandaBreweries: In as much as the taste of apples is refreshing and nice to enjoy, and while the brewery’s first intention is to avail wonderful products for the markets; the product (@tuskerciderug) came with the plight of farmers at the back of our minds*.

## Discussion

Social media, specifically X, is used overwhelmingly by alcohol industry actors in Uganda to promote CSR and alcohol policy framing content. There is little direct product marketing and instead, marketing is more strategic, being orientated towards policy. The framing of policy problems and solutions, and of the actors involved in policymaking and CSR, offers an internally consistent narrative designed to support efforts by alcohol industry actors to secure their preferred alcohol policy outcomes [1]. As the main industry actors are Diageo and AB InBev companies, it is perhaps unsurprising that this narrative closely resembles that used elsewhere, largely successfully to date, in the political strategies of transnational alcohol corporations [46, 47]. The results broadly echo similar studies on alcohol policy developments in Uganda, Nigeria, and South Africa, revealing the alcohol industry’s significant marketing presence and/or dedicated efforts to resist evidence-informed policy reforms [10, 11, 32, 48–54].

The basic tenets of the arguments, such as the emphasis on responsible drinking or drink driving look very familiar because they are longstanding elsewhere [55–57]. More recent content such as COVID as an opportunity has also been seen elsewhere [58–60]. Whilst the structure of the arguments is similar, there is distinct nuance here to be found in the emphases placed on certain content, such as on farmers, illicit trade and contribution to the economy. The significance of the content on partnerships with government and lobbying officials is unclear without access to other data sources. To the extent that the close relationships portrayed accurately reflect alcohol industry-government relationships, there are clearly important public health implications as this entails significant opportunities for alcohol policy interference [61–63].

As elsewhere, what is significant is what this narrative leaves out. It avoids giving attention to the WHO SAFER initiative [64], and measures such as pricing, availability and marketing controls which would make a difference to the levels of alcohol harm endured by Uganda. Rhetorically, X is thus used to create a parallel universe, in which the actual harms and what is known about how to reduce them are conspicuous by their absence [1, 65, 66].

This study can provide specific insights into the goals and strategies of the main alcohol industry actors, though this is constrained due to the limits of this data source and sole reliance on it for this study. This study thus does not have data available on many issues that are relevant to how these actors seek to influence policy in Uganda. For example, we were aware that influencers appeared to play an important role but could not study this phenomenon in-depth. Similarly, we have no data on mass media including newspaper and television content. Achievement of a more comprehensive appreciation of industry actor strategies and goals would need to situate a future study more intimately within the political context and policy developments [2], as well as to pay attention to other political activities that are not promoted, since the actors involved prefer to keep quiet about them [see, for example, 68, 69, 70]. Future studies need to examine also how the contest between industry and public health actors plays out within the policy making process, and trace how formal decisions are influenced by both sets of actors.

There is nonetheless evidence in this study of a division of labour across the industry. The two main brewers focus on burnishing their corporate images with CSR, and prize overt displays of relationship-building. The trade association, on the other hand, addresses policy and regulatory issues directly. Alcohol trade associations elsewhere are under-studied [59, 70–72], and a more advanced literature on the political organisation of the alcohol industry will pay careful attention to the roles played by different organisational types in different policy contexts. Such a literature will be informative more precisely about the circumstances in which industry actors get away with influencing policy, overcoming public health opposition and despite the scientific evidence and conflict of interest in making public health claims, and also when they do not. It will also appreciate the significance of the various components of the political strategies, how they work in synergy or discretely, including across social media and other corporate communications platforms and how they are used to compete directly with public health actors and in other interventions in public policy making.

## Conclusions

Policy framing and CSR promotion by Diageo and AB InBev in Uganda is consistent with industry tactics elsewhere. Alcohol-related harm is presented narrowly, largely limited to illicit trade, drink driving, and underage drinking, with little attention to health. Self-regulation and government partnerships are touted as the most effective policy solutions, with a strong emphasis on personal responsibility. The alcohol industry presents itself as indispensable to Uganda’s future and appears to have developed relationships with politicians and partnerships with the government, as well as having built a coalition with farmers. This means the alcohol industry may be well positioned to oppose the WHO SAFER initiative and the Alcoholic Drinks Control Bill, 2023. This is so, even though their arguments lack substance and are directly at odds with the evidence.

## Data Availability

The datasets used and/or analysed during the current study are available from the corresponding author on reasonable request.

## Abbreviations

CSR: Corporate Social Responsibility
EABL: East African Breweries Limited
UAIA: Uganda Alcohol Industry Association
WHO: World Health Organization
